# Supplementary material for: Biological impact of lead from halide perovskites reveals the risk of introducing a safe threshold
Source: Nat Commun. 2020 Jan 21;11:310. doi: 10.1038/s41467-019-13910-y (PMC6974608; doi:10.1038/s41467-019-13910-y)
Supplement: Supplementary file 1 — Supplementary Information [file 41467_2019_13910_MOESM1_ESM.pdf]

Supporting Information

Biological impact of lead from halide perovskites reveals the risk of introducing a safe threshold

Li et al.

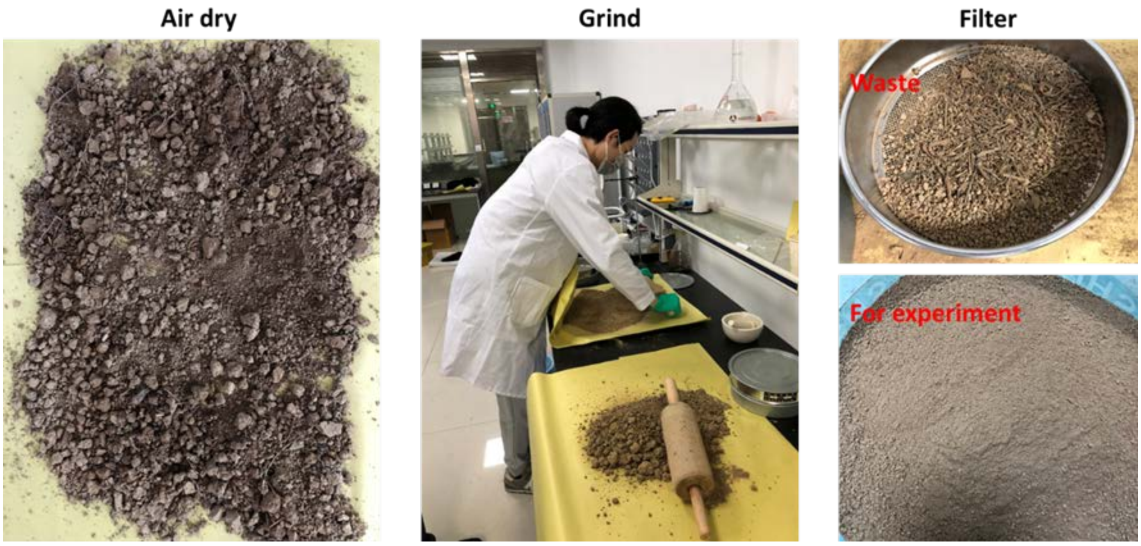

Supplementary Figure 1. The treatments of soil: (a) air-dry, (b) grind, and (c) filter.

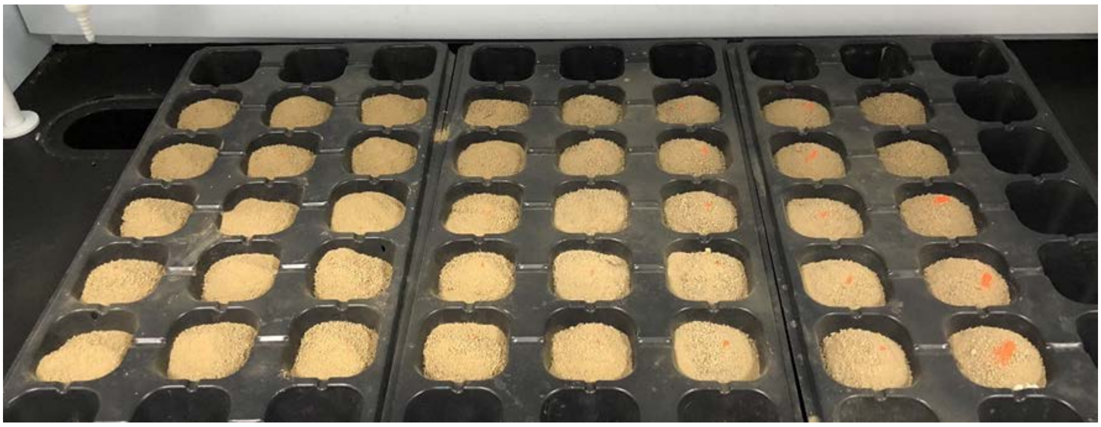

Supplementary Figure 2. The photo of soil in the flowerpots.

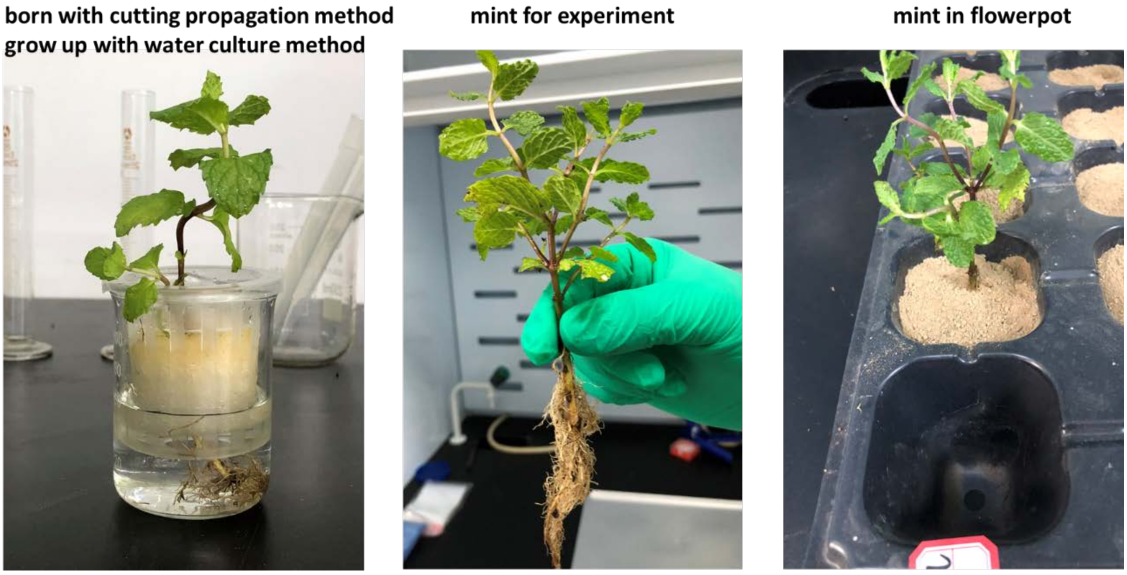

Supplementary Figure 3. The photo of mint; left: born with cutting propagation technique and grown with water culture method; middle: the mint used for the experiment; and right: mint in a flowerpot.

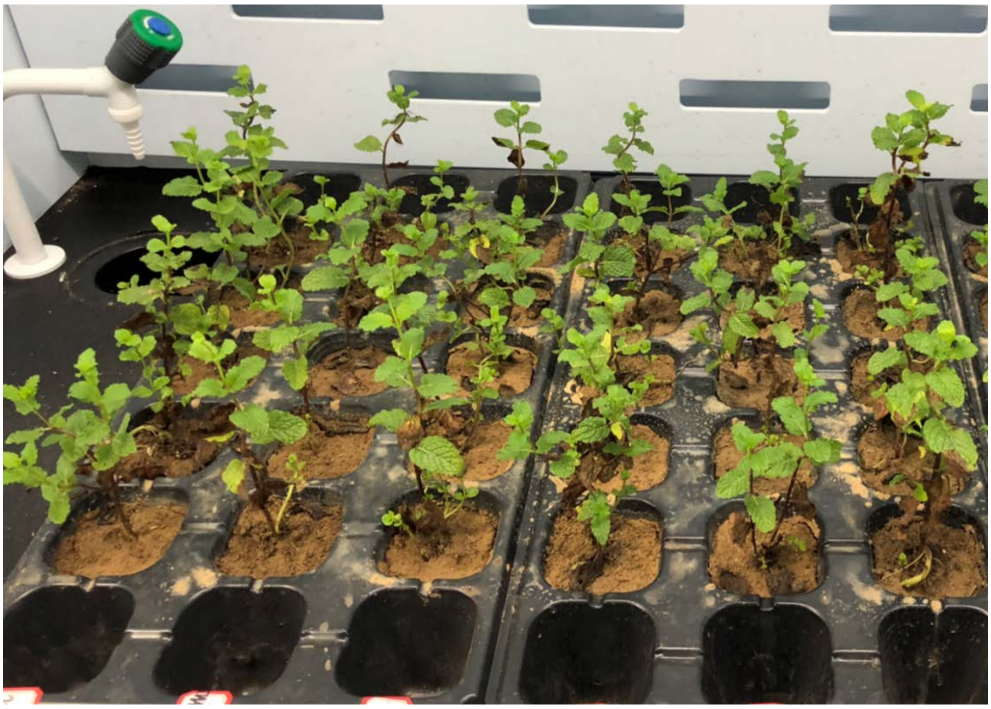

Supplementary Figure 4. A picture of some of the mint plants used in this study.

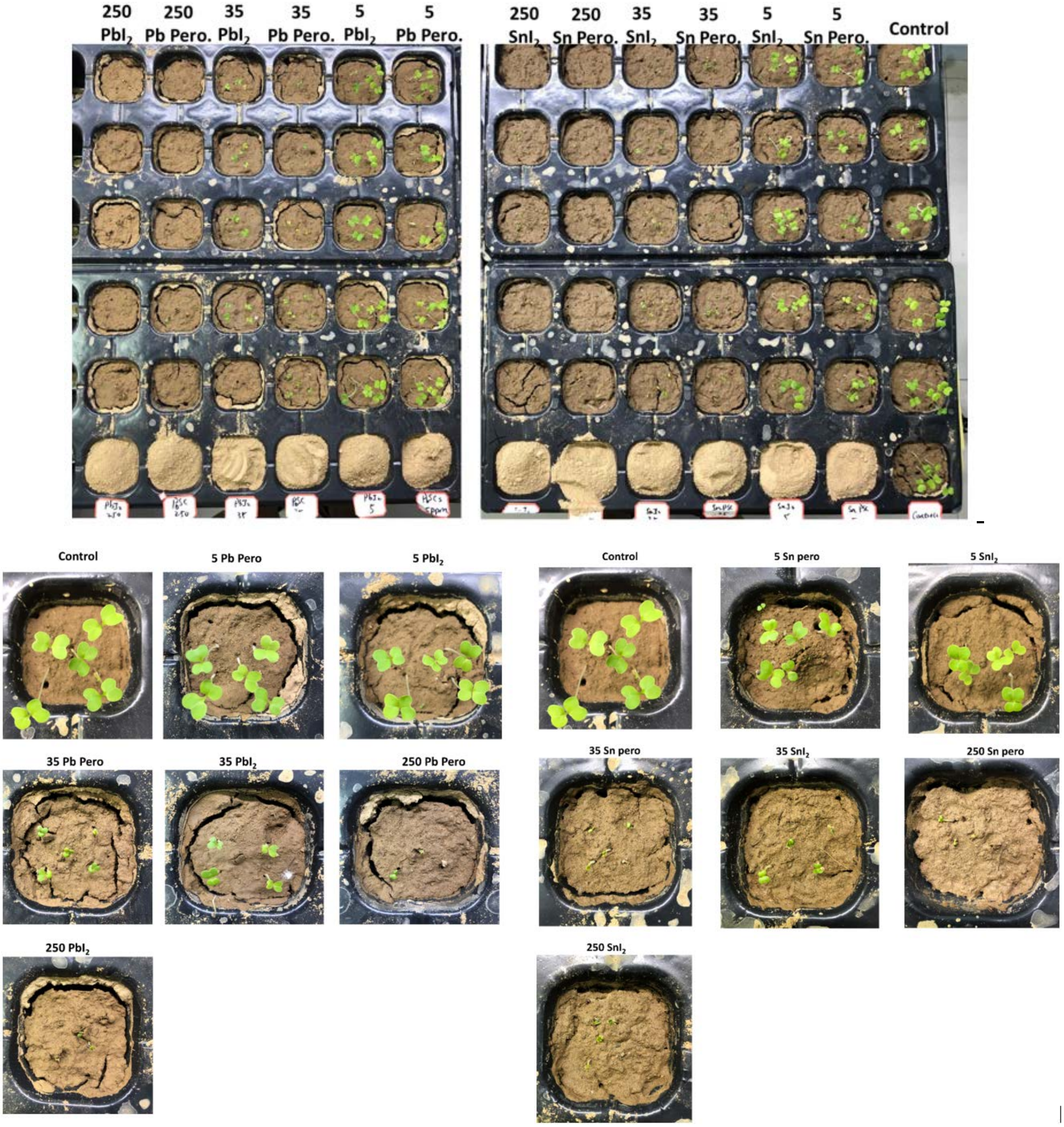

Supplementary Figure 5. Pictures of cabbage seed germination and seedling growth.

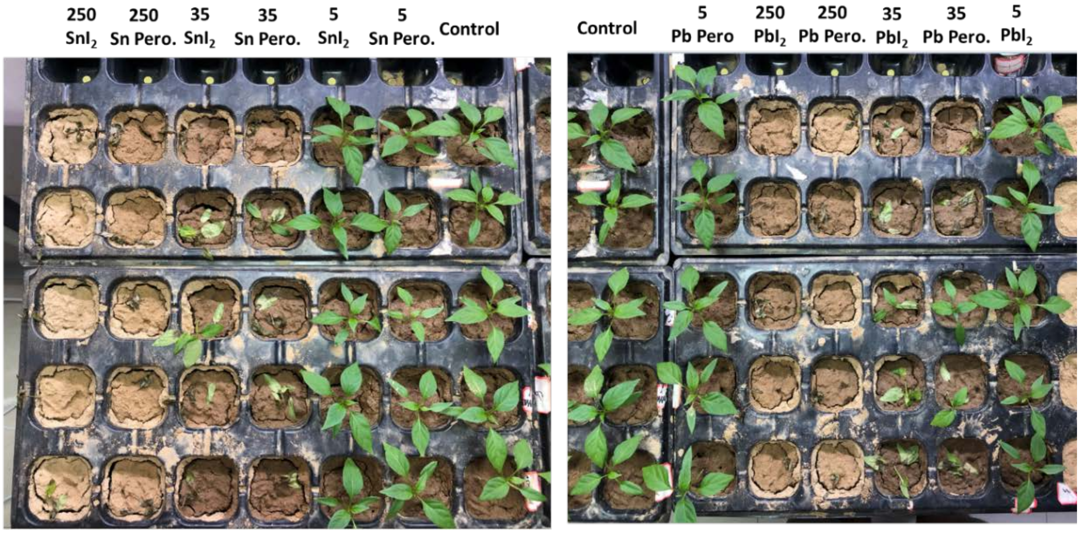

Supplementary Figure 6. Photo of chilli experiment.

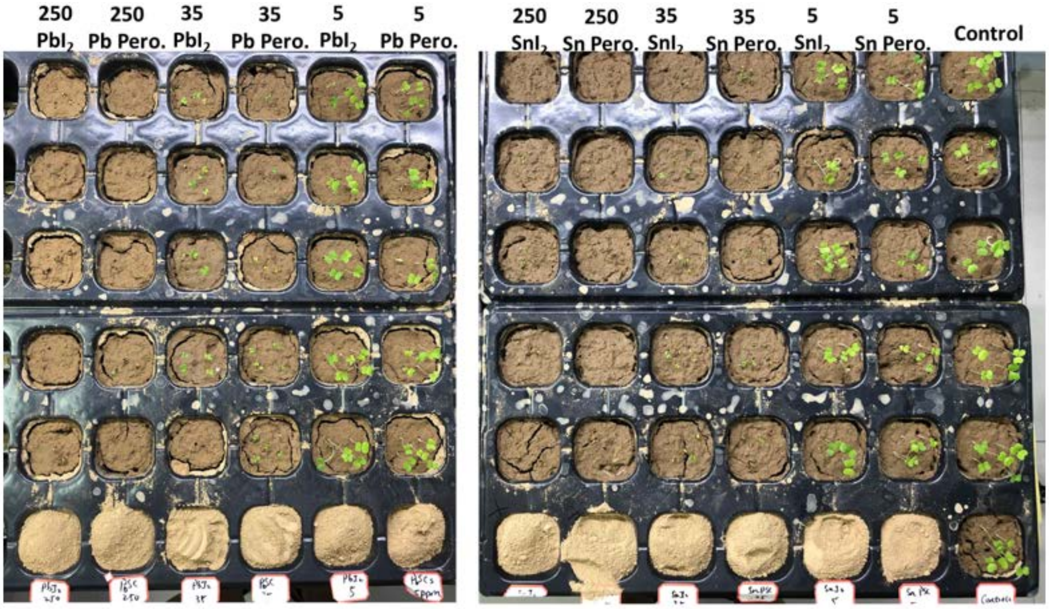

Supplementary Figure 7. Picture of cabbage experiment.

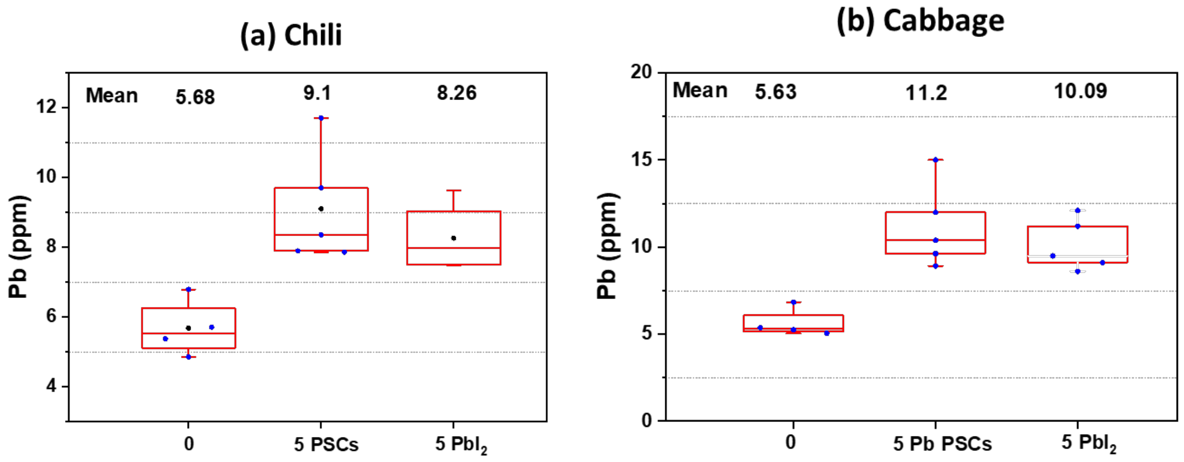

Supplementary Figure 8. Lead concentration in different parts of the plants grown in 5 ppm perovskite (PbI_2+MAI)/PbI_2 contaminated soils and natural soil. Note: to get enough weight for Pb concentration measurement, for the chilli, only roots and stems are used for characterisation; and the whole cabbage plants were used for the Pb concentration characterization.

Original | **Translation**

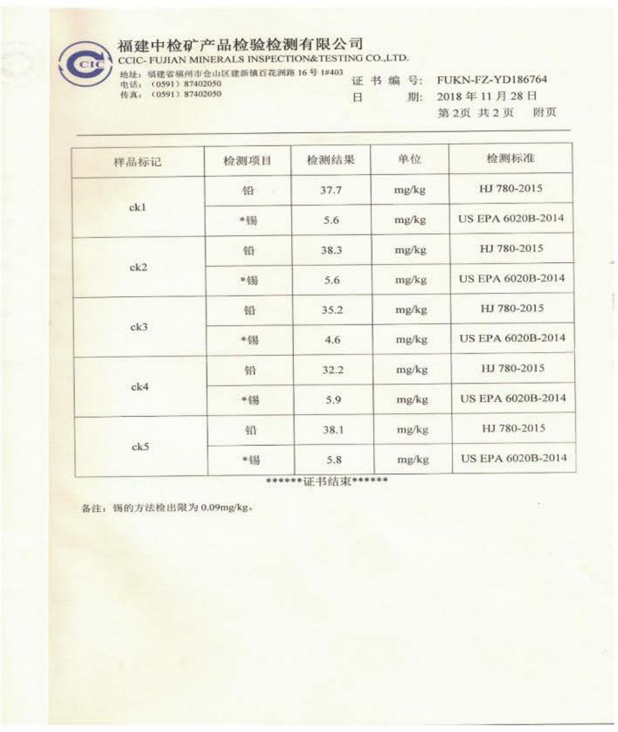

福建中检矿产品检验检测有限公司
CCIC- FUJIAN MINERALS INSPECTION&TESTING CO.,LTD.
地址：福建省福州市仓山区建新镇百花洲路 16 号 1#403
电话：（0591）87402050
传真：（0591）87402050

证 书 编 号：FUKN-FZ-YD186764
日　　　期：2018 年 11 月 28 日
第 2页 共 2 页　附页

样品标记	检测项目	检测结果	单位	检测标准
ck1	铅	37.7	mg/kg	HJ 780-2015
	*锡	5.6	mg/kg	US EPA 6020B-2014
ck2	铅	38.3	mg/kg	HJ 780-2015
	*锡	5.6	mg/kg	US EPA 6020B-2014
ck3	铅	35.2	mg/kg	HJ 780-2015
	*锡	4.6	mg/kg	US EPA 6020B-2014
ck4	铅	32.2	mg/kg	HJ 780-2015
	*锡	5.9	mg/kg	US EPA 6020B-2014
ck5	铅	38.1	mg/kg	HJ 780-2015
	*锡	5.8	mg/kg	US EPA 6020B-2014

******证书结束******

备注：锡的方法检出限为 0.09mg/kg。

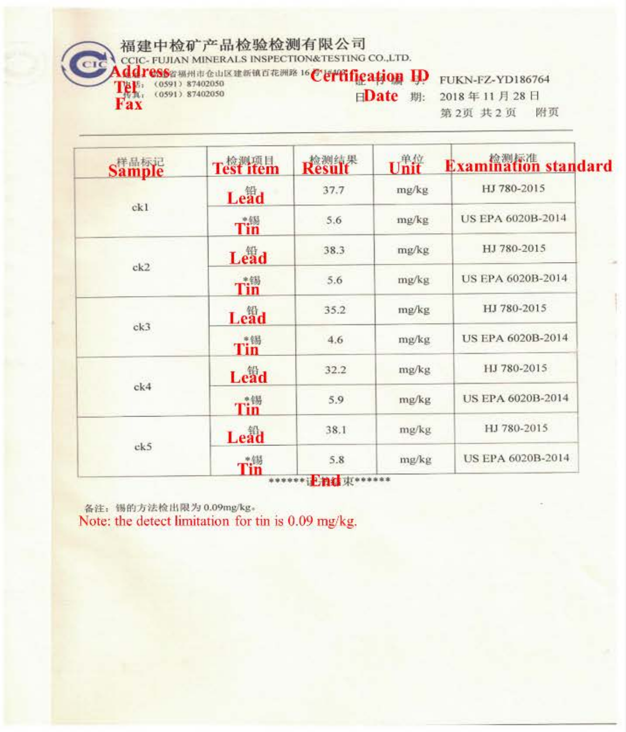

福建中检矿产品检验检测有限公司
CCIC- FUJIAN MINERALS INSPECTION&TESTING CO.,LTD.
Address 地址：福建省福州市仓山区建新镇百花洲路 16 号 1#403
Tel 电话：（0591）87402050
Fax 传真：（0591）87402050

Certification ID 证书编号：FUKN-FZ-YD186764
Date 日期：2018 年 11 月 28 日
第 2页 共 2 页　附页

样品标记 Sample	检测项目 Test item	检测结果 Result	单位 Unit	检测标准 Examination standard
ck1	铅 Lead	37.7	mg/kg	HJ 780-2015
	*锡 Tin	5.6	mg/kg	US EPA 6020B-2014
ck2	铅 Lead	38.3	mg/kg	HJ 780-2015
	*锡 Tin	5.6	mg/kg	US EPA 6020B-2014
ck3	铅 Lead	35.2	mg/kg	HJ 780-2015
	*锡 Tin	4.6	mg/kg	US EPA 6020B-2014
ck4	铅 Lead	32.2	mg/kg	HJ 780-2015
	*锡 Tin	5.9	mg/kg	US EPA 6020B-2014
ck5	铅 Lead	38.1	mg/kg	HJ 780-2015
	*锡 Tin	5.8	mg/kg	US EPA 6020B-2014

******证书结束 End******

备注：锡的方法检出限为 0.09mg/kg。
Note: the detect limitation for tin is 0.09 mg/kg.

Supplementary Figure 9: The lead concentration of natural soil, scanning copy of the certificate by CCIC-FUJIAN MINERALS INSPECTION&TESTING CO., LTD; Left: (a) original and Right: (b) translation.

福建中检矿产品检验检测有限公司

CCIC- FUJIAN MINERALS INSPECTION&TESTING CO.,LTD.

Address：1#403，No.16 BaiHuaZhou Road，Jianxin Town,Cangshan District, Fuzhou,Fujian,P.R.China
Tel：（0591）87402050
Fax：（0591）87402050

Certificate ID: FUKN-FZ-YD186774
Date: March 5, 2019
2/8 Attached page

Sample	Testitem	Result	Unit	Examination standard
a1Y	*Tin	<1.6	mg/kg	Ref.GB 5009.12-2017
a2Y	*Tin	<1.6	mg/kg	Ref.GB 5009.12-2017
a3Y	*Tin	<1.6	mg/kg	Ref.GB 5009.12-2017
a4Y	*Tin	<1.6	mg/kg	Ref.GB 5009.12-2017
a5Y	*Tin	<1.6	mg/kg	Ref.GB 5009.12-2017
o1Y	*Tin	<1.6	mg/kg	Ref.GB 5009.12-2017
o2Y	*Tin	1.6	mg/kg	Ref.GB 5009.12-2017
o3Y	*Tin	3.5	mg/kg	Ref.GB 5009.12-2017
o4Y	*Tin	<1.6	mg/kg	Ref.GB 5009.12-2017
o5Y	*Tin	<1.6	mg/kg	Ref.GB 5009.12-2017

福建中检矿产品检验检测有限公司

CCIC- FUJIAN MINERALS INSPECTION&TESTING CO.,LTD.

Address：1#403，No.16 BaiHuaZhou Road，Jianxin Town,Cangshan District, Fuzhou,Fujian,P.R.China
Tel：（0591）87402050
Fax：（0591）87402050

Certificate ID: FUKN-FZ-YD186774
Date: March 5, 2019
3/8 Attached page

Sample	Testitem	Result	Unit	Examination standard
ü1Y	*Tin	4.8	mg/kg	Ref.GB 5009.12-2017
ü2Y	*Tin	3.7	mg/kg	Ref.GB 5009.12-2017
ü3Y	*Tin	4.4	mg/kg	Ref.GB 5009.12-2017
ü4Y	*Tin	2.9	mg/kg	Ref.GB 5009.12-2017
ü5Y	*Tin	2.6	mg/kg	Ref.GB 5009.12-2017
b1Y	*Tin	2.8	mg/kg	Ref.GB 5009.12-2017
b2Y	*Tin	3.1	mg/kg	Ref.GB 5009.12-2017
b3Y	*Tin	3.5	mg/kg	Ref.GB 5009.12-2017
b4Y	*Tin	<1.6	mg/kg	Ref.GB 5009.12-2017
b5Y	*Tin	2.3	mg/kg	Ref.GB 5009.12-2017
p1Y	*Tin	8.0	mg/kg	Ref.GB 5009.12-2017
p2Y	*Tin	18.7	mg/kg	Ref.GB 5009.12-2017
p3Y	*Tin	6.0	mg/kg	Ref.GB 5009.12-2017
p4Y	*Tin	3.8	mg/kg	Ref.GB 5009.12-2017
p5Y	*Tin	132.0	mg/kg	Ref.GB 5009.12-2017
01Y	*Tin	<1.6	mg/kg	Ref.GB 5009.12-2017
02Y	*Tin	1.6	mg/kg	Ref.GB 5009.12-2017
03Y	*Tin	<1.6	mg/kg	Ref.GB 5009.12-2017
04Y	*Tin	<1.6	mg/kg	Ref.GB 5009.12-2017
05Y	*Tin	<1.6	mg/kg	Ref.GB 5009.12-2017

福建中检矿产品检验检测有限公司

CCIC- FUJIAN MINERALS INSPECTION&TESTING CO.,LTD.

Address：1#403，No.16 BaiHuaZhou Road，Jianxin Town,Cangshan District, Fuzhou,Fujian,P.R.China
Tel：（0591）87402050
Fax：（0591）87402050

Certificate ID: FUKN-FZ-YD186774
Date: March 5, 2019
4/8 Attached page

Sample	Testitem	Result	Unit	Examination standard
a1J	*Tin	<1.6	mg/kg	Ref.GB 5009.12-2017
a2J	*Tin	<1.6	mg/kg	Ref.GB 5009.12-2017
a3J	*Tin	<1.6	mg/kg	Ref.GB 5009.12-2017
a4J	*Tin	2.1	mg/kg	Ref.GB 5009.12-2017
a5J	*Tin	2.8	mg/kg	Ref.GB 5009.12-2017
o1J	*Tin	3.4	mg/kg	Ref.GB 5009.12-2017
o2J	*Tin	4.5	mg/kg	Ref.GB 5009.12-2017
o3J	*Tin	2.9	mg/kg	Ref.GB 5009.12-2017
o4J	*Tin	<1.6	mg/kg	Ref.GB 5009.12-2017
o5J	*Tin	2.4	mg/kg	Ref.GB 5009.12-2017

福建中检矿产品检验检测有限公司

CCIC- FUJIAN MINERALS INSPECTION&TESTING CO.,LTD.

Address：1#403，No.16 BaiHuaZhou Road，Jianxin Town,Cangshan District, Fuzhou,Fujian,P.R.China
Tel：（0591）87402050
Fax：（0591）87402050

Certificate ID: FUKN-FZ-YD186774
Date: March 5, 2019
5/8 Attached page

Sample	Testitem	Result	Unit	Examination standard
ü1J	*Tin	3.8	mg/kg	Ref.GB 5009.12-2017
ü2J	*Tin	8.1	mg/kg	Ref.GB 5009.12-2017
ü3J	*Tin	2.5	mg/kg	Ref.GB 5009.12-2017
ü4J	*Tin	4.3	mg/kg	Ref.GB 5009.12-2017
ü5J	*Tin	1.8	mg/kg	Ref.GB 5009.12-2017
b1J	*Tin	4.4	mg/kg	Ref.GB 5009.12-2017
b2J	*Tin	5.3	mg/kg	Ref.GB 5009.12-2017
b3J	*Tin	7.7	mg/kg	Ref.GB 5009.12-2017
b4J	*Tin	7.5	mg/kg	Ref.GB 5009.12-2017
b5J	*Tin	2.1	mg/kg	Ref.GB 5009.12-2017
p1J	*Tin	21.3	mg/kg	Ref.GB 5009.12-2017
p2J	*Tin	10.2	mg/kg	Ref.GB 5009.12-2017
p3J	*Tin	14.7	mg/kg	Ref.GB 5009.12-2017
p4J	*Tin	13.5	mg/kg	Ref.GB 5009.12-2017
p5J	*Tin	8.2	mg/kg	Ref.GB 5009.12-2017
01J	*Tin	1.6	mg/kg	Ref.GB 5009.12-2017
02J	*Tin	<1.6	mg/kg	Ref.GB 5009.12-2017
03J	*Tin	<1.6	mg/kg	Ref.GB 5009.12-2017
04J	*Tin	<1.6	mg/kg	Ref.GB 5009.12-2017
05J	*Tin	<1.6	mg/kg	Ref.GB 5009.12-2017

福建中检矿产品检验检测有限公司
CCIC- FUJIAN MINERALS INSPECTION&TESTING CO.,LTD.
Address：1#403，No.16 BaiIluaZhou Road，Jianxin Town,Cangshan
District, Fuzhou,Fujian,P.R.China
Tel：（0591）87402050
Fax：（0591）87402050

Certificate ID: FUKN-FZ-YD186774
Date: March 5, 2019
6/8 Attached page

Sample	Testitem	Result	Unit	Examination standard
a1G	*Tin	4.1	mg/kg	Ref.GB 5009.12-2017
a2G	*Tin	<1.6	mg/kg	Ref.GB 5009.12-2017
a3G	*Tin	<1.6	mg/kg	Ref.GB 5009.12-2017
a4G	*Tin	<1.6	mg/kg	Ref.GB 5009.12-2017
a5G	*Tin	<1.6	mg/kg	Ref.GB 5009.12-2017
o1G	*Tin	<1.6	mg/kg	Ref.GB 5009.12-2017
o2G	*Tin	3.3	mg/kg	Ref.GB 5009.12-2017
o3G	*Tin	10.1	mg/kg	Ref.GB 5009.12-2017
o4G	*Tin	2.9	mg/kg	Ref.GB 5009.12-2017
o5G	*Tin	9.6	mg/kg	Ref.GB 5009.12-2017

福建中检矿产品检验检测有限公司
CCIC- FUJIAN MINERALS INSPECTION&TESTING CO.,LTD.
Address：1#403，No.16 BaiIluaZhou Road，Jianxin Town,Cangshan
District, Fuzhou,Fujian,P.R.China
Tel：（0591）87402050
Fax：（0591）87402050

Certificate ID: FUKN-FZ-YD186774
Date: March 5, 2019
7/8 Attached page

Sample	Testitem	Result	Unit	Examination standard
ü1G	*Tin	9.0	mg/kg	Ref.GB 5009.12-2017
ü2G	*Tin	30.8	mg/kg	Ref.GB 5009.12-2017
ü3G	*Tin	13.4	mg/kg	Ref.GB 5009.12-2017
ü4G	*Tin	25.5	mg/kg	Ref.GB 5009.12-2017
ü5G	*Tin	7.8	mg/kg	Ref.GB 5009.12-2017
b1G	*Tin	11.2	mg/kg	Ref.GB 5009.12-2017
b2G	*Tin	11.6	mg/kg	Ref.GB 5009.12-2017
b3G	*Tin	17.9	mg/kg	Ref.GB 5009.12-2017
b4G	*Tin	9.9	mg/kg	Ref.GB 5009.12-2017
b5G	*Tin	9.5	mg/kg	Ref.GB 5009.12-2017
p1G	*Tin	93.5	mg/kg	Ref.GB 5009.12-2017
p2G	*Tin	37.5	mg/kg	Ref.GB 5009.12-2017
p3G	*Tin	76.4	mg/kg	Ref.GB 5009.12-2017
p4G	*Tin	71.3	mg/kg	Ref.GB 5009.12-2017
p5G	*Tin	60.5	mg/kg	Ref.GB 5009.12-2017
01G	*Tin	<1.6	mg/kg	Ref.GB 5009.12-2017
02G	*Tin	<1.6	mg/kg	Ref.GB 5009.12-2017
03G	*Tin	<1.6	mg/kg	Ref.GB 5009.12-2017
04G	*Tin	<1.6	mg/kg	Ref.GB 5009.12-2017
05G	*Tin	<1.6	mg/kg	Ref.GB 5009.12-2017

福建中检矿产品检验检测有限公司
CCIC- FUJIAN MINERALS INSPECTION&TESTING CO.,LTD.
Address：1#403，No.16 BaiHuaZhou Road，Jianxin Town,Cangshan
District, Fuzhou,Fujian,P.R.China
Tel：（0591）87402050
Fax：（0591）87402050

Certificate ID: FUKN-FZ-YD186774
Date: March 5, 2019
8/8 Attached page

Sample	Testitem	Result	Unit	Examination standard
soil01	effective lead	7.36	mg/kg	HJ 804-2016
soil02	effective lead	7.17	mg/kg	HJ 804-2016
soil03	effective lead	7.16	mg/kg	HJ 804-2016
soil04	effective lead	7.14	mg/kg	HJ 804-2016
soil05	effective lead	7.38	mg/kg	HJ 804-2016

******END******

Remarks: the detection limit of tin in plants was 1.6mg/kg.

Supplementary Figure 10: Tin concentration of plants, scanning copy of the certificate from CCIC-FUJIAN MINERALS INSPECTION&TESTING CO., LTD.

Supplementary Table 1: Pb concentration in natural soil in this experiment

Sample	01	02	03	04	05	Mean
**Concentration (mg kg^{-1})**	37.7	38.3	35.2	32.2	38.1	36.3

Supplementary Table 2: Environmental quality standards for lead in soil of different countries

Country	China	Australia	Canada	France	Germany	Japan	Netherlands	UK	USA
Values (ppm)	250	100	140	400	400	150	530	450	400

Supplementary Table 3: Pb concentration in 5-ppm lead perovskite contaminated soil

**Sample**	**01**	**02**	**03**	**04**	**05**	**Mean**
**Concentration ($mg\ kg^{-1}$)**	40.8	40.1	41.3	39.1	41.9	40.6

Supplementary Table 4. Lead concentration in the 30-days old mint used for the experiment.

**Sample**	**01**	**02**	**03**	**04**	**05**	**Mean**
**ROOTS ($mg\ kg^{-1}$)**	1.8	0.4	0.3	0.9	2.0	1.1
**STEMS ($mg\ kg^{-1}$)**	1.0	0.9	1.7	/	0.5	0.5
**LEAVES ($mg\ kg^{-1}$)**	0.7	0.4	0.2	0.4	0.4	0.4

Supplementary Table 5. Tin concentration in 30-days old mint used for the experiment

Sample	01	02	03	04	05	Mean
**ROOTS (mg kg^{-1})**	<1.6	<1.6	<1.6	<1.6	<1.6	<1.6
**STEMS (mg kg^{-1})**	1.6	<1.6	<1.6	<1.6	<1.6	<1.6
**LEAVES (mg kg^{-1})**	<1.6	<1.6	<1.6	<1.6	<1.6	<1.6

* The detect limitation for tin concentration is 1.6 mg kg^{-1}.

Supplementary Table 6: Pb uptake by mint plants grown in Pb perovskite contaminated soils

**PbI_2+MAI**	**ROOT (mg kg^{-1})**		**STEM (mg kg^{-1})**		**LEAF (mg kg^{-1})**	
		average		average		average
250 mg kg^{-1}	4896.8	3905.83	178.99	209.55	426.79	405.73
	3416.6		240.11		384.67	
	3404.1		/		/	
35 mg kg^{-1}	119.51	130.39	6.73	10.53	/	/
	141.87		13.61		/	
	129.54		11.26		/	
5 mg kg^{-1}	28.77	26.85	4.13	4.69	8.82	9.97
	24.86		4.43		10.34	
	26.92		5.51		10.76	
Control (0 mg kg^{-1})	9.32	11.99	3.88	3.36	8.44	7.97
	11.49		2.84		7.5	
	15.16		/		/	

Supplementary Table 7: Pb uptake by mint plants grown in 5-ppm Pb perovskite and PbI_2 contaminated soils

	**ROOT (mg kg^{-1})**		**STEM (mg kg^{-1})**		**LEAF (mg kg^{-1})**	
		average		average		average
Control (0 mg kg^{-1})	9.32	11.99	3.88	3.36	8.44	7.97
	11.49		2.84		7.5	
	15.16		/		/	
PbI_2+MAI	28.77	26.85	4.13	4.69	8.82	9.97
	24.86		4.43		10.34	
	26.92		5.51		10.76	
PbI_2	22.61	22.44	2.49	2.72	6.65	5.97
	19.84		2.66		6.14	
	24.9		3.03		5.13	

Supplementary Table 8: Sn uptake by mint plants grown in Sn perovskite contaminated soils

SnI_2+MAI	ROOT (mg kg^{-1})		STEM (mg kg^{-1})		LEAF (mg kg^{-1})	
		average		average		average
250	93.5	67.8	21.3	13.6	8.0	9.1
	37.5		10.2		18.7	
	76.4		14.7		6.0	
	71.3		13.5		3.8	
	60.5		8.2		132.0	
35	9.0	17.3	3.8	4.5	4.8	3.7
	30.8		8.1		3.7	
	13.4		2.5		4.4	
	25.5		4.3		2.9	
	7.8		1.8		2.6	
5	<1.6	6.5	3.4	3.6	<1.6	2.5
	3.3		4.5		1.6	
	10.1		2.9		3.5	
	2.9		<1.6		<1.6	
	9.6		2.4		<1.6	
Control	4.1	4.1	<1.6	2.4	<1.6	<1.6
	<1.6		<1.6		<1.6	
	<1.6		<1.6		<1.6	
	<1.6		2.1		<1.6	
	<1.6		2.8		<1.6	

Supplementary Table 9: Sn uptake by mint plants grown in 35-ppm Sn perovskite and SnI_2 contaminated soils

	ROOT (mg kg^{-1})		STEM (mg kg^{-1})		LEAF (mg kg^{-1})	
		average		average		average
SnI_2+MAI	9.0	17.3	3.8	4.5	4.8	3.7
	30.8		8.1		3.7	
	13.4		2.5		4.4	
	25.5		4.3		2.9	
	7.8		1.8		2.6	
SnI_2	11.2	12.1	4.4	5.4	2.8	2.9
	11.6		5.3		3.1	
	17.9		7.7		3.5	
	9.9		7.5		<1.6	
	9.5		2.1		2.3	
Control	4.1	4.1	<1.6	2.4	<1.6	<1.6
	<1.6		<1.6		<1.6	
	<1.6		<1.6		<1.6	
	<1.6		2.1		<1.6	
	<1.6		2.8		<1.6	

Supplementary Table 10. Pb concentration in chilli

**Pb^{2+} added ($mg\ kg^{-1}$)**	**ROOTS + STEMS ($mg\ kg^{-1}$)**	
	PbI_2	PbI_2+MAI
**5**	7.48	11.7
	9.62	8.35
	8.44	7.86
	7.49	9.70
	N/A	7.89
**Control**	6.79	
	5.71	
	4.85	
	5.37	

Supplementary Table 11. Pb concentration in cabbage

**Pb^{2+} added (mg kg^{-1})**	**Pb in plant (mg kg^{-1})**	
	PbI_2	PbI_2+MAI
**5**	9.11	15.0
	9.49	8.92
	11.2	10.4
	12.1	12.0
	8.60	9.63
	6.83	/
**Control**	5.26	
	5.37	
	5.05	
	9.11	

Supplementary Note 1. Perovskite amount added to the soil

The thickness of (methylammonium lead iodide) $MAPbI_3$ layer in the PSCs is around 600 nm. Considering that the $MAPbI_3$ density is 4.09 g cm^{-3} and the lead content in $MAPbI_3$ is 33.4% in weight, a PSC module would contain 0.82 g of lead per square meter. For the surface soil (0 to 15 cm depth), considering a density between 1.3 and 1.4 g/cm^3, the soil density is in the range of 1.95×10^5 to 2.1×10^5 g per square meters. If it leakages to an equivalent area of soil, the lead concentration will increase by 3.9 – 4.2 mg kg^{-1}. We use an average value of 4 mg kg^{-1}.

Pb^{2+} experiments:

• 5 mg kg^{-1} Pb^{2+} in perovskite ($MAI+PbI_2$): add 0.375 mg MAI and 2.22 mg PbI_2 into the 100 g natural soil.

• 35 mg kg^{-1} Pb^{2+} in perovskite ($MAI+PbI_2$): add 3.68 mg MAI and 15.54 mg PbI_2 into the 100 g natural soil.

• 250 mg kg^{-1} Pb^{2+} in perovskite ($MAI+PbI_2$): add 18.75 mg MAI and 111 mg PbI_2 into the 100 g natural soil.

• 5 mg kg^{-1} Pb^{2+} in PbI_2: add 2.22 mg PbI_2 into the 100 g natural soil.

• 35 mg kg^{-1} Pb^{2+} in PbI_2: add 15.54 mg PbI_2 into the 100 g natural soil.

• 250 mg kg^{-1} Pb^{2+} in PbI_2: add 111 mg PbI_2 into the 100 g natural soil.

Sn^{2+} experiments:

• 5 mg kg^{-1} Sn^{2+} in perovskite ($MAI+SnI_2$): add 0.67 mg MAI and 1.56 mg SnI_2 into the 100 g natural soil.

• 35 mg kg^{-1} Sn^{2+} in perovskite ($MAI+SnI_2$): add 4.70 mg MAI and 10.94 mg SnI_2 into the 100 g natural soil.

• 250 mg kg-1 Sn^{2+} in perovskite ($MAI+SnI_2$): add 33.5 mg MAI and 78.0 mg SnI_2 into the 100 g natural soil.

• 5 mg kg^{-1} Sn^{2+} in SnI_2: add 1.56 mg SnI_2 into the 100 g natural soil.

• 35 mg kg^{-1} Sn^{2+} in SnI_2: add 10.94 mg SnI_2 into the 100 g natural soil.

• 250 mg kg^{-1} Sn^{2+} in SnI_2: add 78.0 mg SnI_2 into the 100 g natural soil.

Supplementary Note 2. Soil treatment

The soil was collected from agricultural land (managed and protected by the university) in Fujian Agriculture and Forestry University, China (latitude 26.084, longitude 119.238). The Pb/Sn exist in natural soil in several speciations: exchangeable, carbonate bounded, oxides, organic, and residual form. The soil samples were firstly air-dried about ten days to remove the moisture content; then the soil samples were grinded and filtered (2-mm-filter) to remove the sundries, as shown in Supplyment Figure 1. We measured the Pb concentration from five soil samples: which were 37.7, 38.3, 35.2, 32.2, and 38.1 mg kg^{-1}, respectively; thus the mean value was 36.3 mg kg^{-1} (Supplementary Table 1). In China, where the more significant production of lead is concentrated, the agricultural regulation tolerates a lead content into the soil up to 250 mg kg^{-1}; worldwide the maximum lead concentration in the land for agriculture depends on the law of the country, as shown in Supplementary Table 2 for some of them.

The fundamental chemical reaction for perovskites (for example, $MAPbI_3$) is reversible, as follows:

$$PbI_2\ (s) + MAI\ (aq) \leftrightarrow MAPbI_3\ (s) \quad (1)$$

In the presence of oxygen, water and moisture, the chemical equilibrium can drive the reaction towards the precursors, leading to the decomposition of $MAPbI_3$ into MAI and PbI_2 [1-4]. Thus, for simplicity, we mixed the MAI and PbI_2 power directly in the soil. In this study, both MAI+PbI_2 and PbI_2 were added into an independent flowerpot (as shown in Supplementary Figure 2). Then, the soil was stirred 50 times with a spatula. We measured the lead concentration for the 5-ppm lead perovskite contaminated soil to check the distribution of the lead. The data shown in Supplementary Table 3 displays that the lead is homogeneously distributed in the soil sample.

Supplementary Note 3. *Capsicum Annuum* and *Brassica Campestris* as alternatives of mint plants

The metal uptake by plants is influenced by soil pH, the metal concentration in the soils, metal cation exchange capability between roots and soil, and other physochemical factors [c]. We have been using mint as a representative plant for our study since mint is a rustic hyper-tolerant plant to several heavy metals (lead, tin, thallium, etc.). [5,6]

Besides the mint plants experiment, we performed additional analysis based on *Capsicum Annuum* (chilli), which is a low Pb accumulating capability plant and *Brassica Campestris* (cabbage), which is a middle Pb accumulating capability plant. Most of the chilli and cabbage died at 35 and 250 ppm (as shown in Supplementary Figure 6 and 7). For the survived plants, both the chilli and cabbage show the same trends as the results obtained from the mint plants.

For the chilli: After adding 5 mg kg^{-1} Pb^{2+} perovskite in soil, the mean lead concentration in mint roots and stems showed a significant increase up to 9.1 mg kg^{-1}. Considering that the lead concentration in natural soil was 36.3 mg kg^{-1}, which means we only add extra 13.8% Pb^{2+} in native soil, but the lead concentration increased about 60% compared to that grown in natural soil (as shown in Supplementary Figure 8).

For the cabbage: After adding 5 mg kg^{-1} Pb^{2+} perovskite in soil, the mean lead concentration in cabbage plants showed a significant increase up to 11.2 mg kg^{-1}. Considering the lead concentration in natural soil is 36.3 mg kg^{-1}, which means we only add extra 13.8% Pb^{2+} in natural soil, but the lead concentration increased up to 98.9% than in plants grown in native soil (as shown in Supplementary Figure 8).

Supplementary Note 4. Mint plants grow

Thirty days old *Mentha Spicata* plants, which were born with cutting propagation technique and grown with water culture method, were purchased from Qingdao Baicaoxiang Fangxiang plants Co. Ltd. We have measured the lead/tin concentration in the 30 days old mint before to use them for our experiments, and we found that the lead and tin concentration was significantly lower than the value we collected during the study (see Supplementary Table 2 and Table 3). The plants were grown in the 8 cm thick soil for 20 days. During the growth, the mint roots were fully immersed into the soil.

Five plants were grown each in 100 g of soils. During the growth stages, the plants were watered with 10 mL of de-ionised water (>18 M, without any metal cations) every two days. There was no drainage system (as shown in Supplementary Figure 3 and 4). During the growth, the temperature was settled as 20 °C. Every day, the plants were illuminated with 104 lux light for 16 hours and other 8 hours in the dark. After harvest, they were rinsed with de-ionised water and ultra-sonicated for 2 min to remove the adhering soil particles and dust. Then mint roots, stems and leaves were separated and dried at 60 °C in an oven for 48 hours. Then the samples were chopped into pieces, and a representative sample of 0.1 g was taken for metal concentration measurement by inductively coupled plasma mass spectrometry (ICP-MS, Agilent Technology 7700 series).

Supplementary Note 5. Seed germination and early seedling growth

The mint plants were born with cutting propagation technique and grown with water culture method; thus, we can’t investigate the influence of Pb/Sn leakage to the early development of the plants. We perform and additional experiment based on *Brassica Campestris* (cabbage) from seeds sown. We found that both lead and tin cause inability of seeds to germinate and reduce the seed growth. In the 35 and 250 ppm, Pb/Sn contaminated soil, the seed germination and seed growth were sharply reduced compared to control, as visible from Supplementary Figure 5. Moreover, seed growth is much slower in Sn cations contaminated soil. This could be because the Sn is known for its higher ability to acidify than Pb [b].

Supplementary Refernces

1 CHEN, S.-b., WANG, M., LI, S.-s. & ZHAO, Z.-q. Overview on current criteria for heavy metals and its hint for the revision of soil environmental quality standards in China. *J. Integr.* **17**, 765-774 (2018).

2 Kjaer, C., Pedersen, M. & Elmegaard, N. Effects of soil copper on black bindweed (Fallopia convolvulus) in the laboratory and in the field. *ARCH ENVIRON CON TOX* **35**, 14-19 (1998).

3 Zeng, F. *et al.* The influence of pH and organic matter content in paddy soil on heavy metal availability and their uptake by rice plants. *Environ. Pollut.* **159**, 84-91 (2011).

4 Zheljazkov, V. D., Craker, L. E. & Xing, B. Effects of Cd, Pb, and Cu on growth and essential oil contents in dill, peppermint, and basil. *ENVIRON EXP BOT* **58**, 9-16 (2006).

5 Ghasemidehkordi, B. *et al.* Tin Levels in Perennial and Annual Green Leafy Vegetables. *Int. J. Veg. Sci.* **23**, 340-345 (2017).

6 Prasad, A., Singh, A. K., Chand, S., Chanotiya, C. & Patra, D. Effect of chromium and lead on yield, chemical composition of essential oil, and accumulation of heavy metals of mint species. *COMMUN SOIL SCI PLAN* **41**, 2170-2186 (2010).
